# Screening Strategies for Tuberculosis Prevalence Surveys: The Value of Chest Radiography and Symptoms

**DOI:** 10.1371/journal.pone.0038691

**Published:** 2012-07-06

**Authors:** Anna H. van’t Hoog, Helen K. Meme, Kayla F. Laserson, Janet A. Agaya, Benson G. Muchiri, Willie A. Githui, Lazarus O. Odeny, Barbara J. Marston, Martien W. Borgdorff

**Affiliations:** 1 Kenya Medical Research Institute, Kenya Medical Research Institute/Center for Disease Control Research and Public Health Collaboration, Kisumu, Kenya; 2 Academic Medical Centre, University of Amsterdam, Amsterdam, The Netherlands; 3 Kenya Medical Research Institute, Centre for Respiratory Diseases Research, Nairobi, Kenya; 4 Centers for Disease Control and Prevention, Center for Global Health, Atlanta, Georgia, United States of America; Hopital Raymond Poincare - Universite Versailles St. Quentin, France

## Abstract

**Background:**

We conducted a tuberculosis (TB) prevalence survey and evaluated the screening methods used in our survey, to assess if screening in TB prevalence surveys could be simplified, and to assess the accuracy of screening algorithms that may be applicable for active case finding.

**Methods:**

All participants with a positive screen on either a symptom questionnaire, chest radiography (CXR) and/or sputum smear microscopy submitted sputum for culture. HIV status was obtained from prevalent cases. We estimated the accuracy of modified screening strategies with bacteriologically confirmed TB as the gold standard, and compared these with other survey reports. We also assessed whether sequential rather than parallel application of symptom, CXR and HIV screening would substantially reduce the number of participants requiring CXR and/or sputum culture.

**Results:**

Presence of any abnormality on CXR had 94% (95%CI 88–98) sensitivity (92% in HIV-infected and 100% in HIV-uninfected) and 73% (95%CI 68–77) specificity. Symptom screening combinations had significantly lower sensitivity than CXR except for ‘any TB symptom’ which had 90% (95%CI 84–95) sensitivity (96% in HIV-infected and 82% in HIV-uninfected) and 32% (95%CI 30–34) specificity. Smear microscopy did not yield additional suspects, thus the combined symptom/CXR screen applied in the survey had 100% (95%CI 97–100) sensitivity. Specificity was 65% (95%CI 61–68). Sequential application of first a symptom screen for ‘any symptom’, followed by CXR-evaluation and different suspect criteria depending on HIV status would result in the largest reduction of the need for CXR and sputum culture, approximately 36%, but would underestimate prevalence by 11%.

**Conclusion:**

CXR screening alone had higher accuracy compared to symptom screening alone. Combined CXR and symptom screening had the highest sensitivity and remains important for suspect identification in TB prevalence surveys in settings where bacteriological sputum examination of all participants is not feasible.

## Introduction

Tuberculosis (TB) prevalence surveys are the most direct tool to measure the TB burden in a population and monitor the performance of TB control programs in areas where routine surveillance systems are weak [1], [2], but are logistically challenging and costly. TB prevalence surveys are strongly recommended in 21 global focus countries, of which 12 are in Africa where national surveys have rarely been undertaken in the last 50 years. [2].

The most accurate measure of the prevalence of bacteriologically confirmed pulmonary TB is achieved if multiple sputum cultures are performed of all participants. For large surveys, sufficient laboratory capacity may not be available, and the costs for this approach may be prohibitive. [1], [3], [4] Therefore, a screening step is often used to select participants for culture examination. For prevalence surveys, the ideal screening algorithm balances accuracy and repeatability with feasibility. High sensitivity limits the underestimation of prevalence, high specificity limits the burden and costs of sputum cultures, and high repeatability allows comparison with consecutive surveys or other regions. Reported screening tools include symptom questionnaires [5], [6], [7], chest radiography [8], sputum culture [9], sputum microscopy and combinations of these. [10] TB screening tools are also applied to improve early case detection and reduce TB transmission. [11].

We conducted a TB prevalence survey in western Kenya, in which all participants were examined by symptom questionnaire, CXR and sputum microscopy. [12] If any one of these screening methods were positive, sputum for culture was obtained. In this report, we evaluated the screening methods used in our survey with the aim to: (1) assess if screening in TB prevalence surveys could be simplified, and (2) assess the accuracy of screening algorithms that may be applicable for active case finding. We also compared our results with reports from the literature.

## Methods

### Ethical Approval

The prevalence survey protocol was approved by the Kenya Medical Research Institute Scientific Steering Committee and Ethics Review Committee (protocol number 943), and by the US Centers for Disease Control and Prevention Institutional Review Board (IRB-G; protocol number 4712). Written informed consent was obtained of the survey participants.

### Survey

We conducted a TB prevalence survey between August 2006 and December 2007 in the Asembo (Rarieda District) and Gem District areas in western Kenya, where HIV prevalence was 14.9% in persons 15–64 years old. [12] The detailed methods and main results have been described elsewhere. [13] In short, after individual written informed consent was obtained at the homes, a questionnaire was administered, using handheld computers, with questions on the presence and duration of symptoms that are possibly suggestive of TB (cough, haemoptysis, weight loss, fever, night sweats). Duration of cough less than 2 weeks was recorded in days, and of 2 weeks and longer in weeks. Symptoms qualifying as a positive symptom screen were the presence of cough for more than 7 days, and/or haemoptysis of any duration and/or two out of three of the following symptoms: fever for >7 days, night sweats for >7 days, or weight loss resulting in a changed fit of clothes. We requested all participants to provide two sputum samples for fluorescence smear microscopy, and to have one posterior-anterior chest radiograph (CXR) taken in a mobile unit at a nearby location, using conventional x-ray technology and automatic film processing. One additional sputum sample for mycobacterial culture (Löwenstein-Jensen slants [14], and MGIT™ Manual Mycobacterial Growth System [Becton Dickinson, Franklin Lakes, USA]) and microscopy was requested at the CXR location from survey participants who either had an abnormal CXR (any abnormality) identified during field reading by clinical officers [15] and/or a positive symptom screen, or at a later time if sputum smear microscopy was positive. Participants who qualified for sputum culture examination after screening are hereafter referred to as suspects. Participants who did not qualify as a suspect were considered not to have TB, which implies that asymptomatic persons with smear-negative culture positive TB but normal CXR were missed. From the literature we estimate this to be less than 5% [16], [17]. CXR reading was recorded on scannable forms.

A case of bacteriologically confirmed pulmonary TB (PTB) was defined by either one culture positive for *M tuberculosis*, or two sputum smears positive for acid fast bacilli not explained by a culture positive for nontuberculous mycobacteria. HIV testing was offered to participants with confirmed PTB. The final analysis included 20,566 residents aged 15 years and above from 40 sampled clusters. Of the 123 persons identified with pulmonary TB, 47 (38%) were smear-positive, culture-positive, 72 (59%) smear-negative, culture-positive, and 4 (3%) smear-positive, culture negative. The prevalence of bacteriologically confirmed TB was 6.0 per 1000 (95% confidence interval (CI) 4.6–7.4), and of smear-positive TB 2.5 per 1000 (95%CI 1.6–3.4). HIV prevalence among confirmed TB cases was 51%. [13].

### Screening Strategies and Statistical Analysis

We examined the association between each of the symptoms and TB in single variable analysis and in a multiple logistic regression model. We defined screening strategies (described in Table 1) of combinations of symptoms and/or CXR abnormalities that would be simplifications of the strategy applied in our survey or, to allow comparison, resemble screening strategies used in other prevalence surveys [9], [10], [18], [19] or TB case finding situations. [20] We calculated the sensitivity, specificity, predictive values with 95% confidence intervals (CIs), and the area under the receiver operating characteristics curve (AUC) as a summary measure of diagnostic accuracy of each screening strategy, using bacteriologically confirmed TB as the reference standard. We stratified the calculation of sensitivity by HIV status.

**Table 1:** Description of Screening Strategies.

*Symptom combinations*	
*1.*	Cough ≥2 weeks
*2.*	Any symptom (cough, haemoptysis, fever, night sweats, weight loss) of any duration or severity
*3.*	Cough ≥3 weeks or haemoptysis
*4.*	Productive cough ≥2 weeks
*5.*	Cough ≥2 weeks or weight loss
*6.*	Symptom combination used in this survey: Cough >7 days, and/or haemoptysis and/or ≥2 out of the following symptoms: fever (for >7 days), night sweats (for >7 days), weight loss resulting in a changed fit of clothes
*Symptom and smear microscopy*	
*7.*	Cough ≥2 weeks and smear positive (≥ scanty [1–9 AFB/whole smear])
*Chest radiography*	
*8.*	Any abnormality
*9.*	Pulmonary and/or pleural abnormalities only
*Combinations (applied in parallel)*	
*10.*	Screening strategy used in this survey: Any abnormality on CXR and/or positive symptom screening as in #6 above
*11.*	Any abnormality on CXR and/or cough ≥2 weeks
*12.*	Any abnormality on CXR and/or cough >7 days (systemic symptoms excluded)
*13.*	Any abnormality on CXR and/or cough ≥2 weeks (rather than >7 days) and/or haemoptysis and/or ≥2 out of: fever (for >7 days), night sweats (for >7 days), weight loss (changed fit of clothes)
*14.*	Pulmonary and/or pleural abnormality on CXR and/or cough ≥2 weeks and/or ≥2 out of: fever (for >7 days), night sweats (for >7 days), weight loss (changed fit of clothes)
*Sequential combination taking HIV-status into account*	
*15.*	Step 1: Symptoms screening first. If positive for any symptom of any duration, CXR is taken.Step 2: If HIV-negative, only persons with a CXR abnormality are suspected of TB. If HIV-positive, persons with either a CXR abnormality or a positive symptom combination as in #10 are suspected of TB.

The numbers are referred to in the text, tables and figure.

In addition we assessed whether the proportion of participants requiring sputum culture, CXR or symptom interview in prevalence surveys could be reduced by sequential (rather than parallel) application of the same symptom combination and CXR screening (any abnormality) as used in our survey, in different sequences. Participants with a positive first screen would then qualify as a suspect from whom sputum for culture would be collected, and not be further subjected to the second screen. We explored the scenario whereby participants would be interviewed for presence of symptoms first, and only if they would have a negative symptom screen receive CXR, Or, vice versa, participants would first be invited for CXR, and only be interviewed for symptoms if the CXR were normal.

Lastly we explored scenarios in which participant’s HIV status would be considered as part of the suspect criteria, and all participants would first be screened for symptoms, but the asymptomatic participants would not be evaluated further. We report a scenario where participants who had any symptom (of any duration or severity) would undergo CXR. HIV-uninfected persons with any symptom would only be considered to be TB suspects if a CXR abnormality were present. In HIV-infected participants, CXR and symptoms would be further considered and they would be TB suspects if either a CXR abnormality were present, and/or their symptoms were suggestive for TB as defined by the survey symptom combination (i.e. the presence of cough for more than 7 days, and/or haemoptysis or two out of three of fever (present for >7 days), night sweats (present for >7 days), weight loss resulting in a changed fit of clothes).

Data were analyzed with SAS 9.2 survey procedures (SAS Institute Inc., Cary, North Carolina, USA), which takes correlation within the cluster into account using the Taylor series (linearization) method, and for associations uses the Rao-Scott Wald chi-square statistic to adjust for cluster design in bivariate analysis and logistic regression. The 95% confidence intervals (CIs) of the sensitivity, specificity, and predictive values were adjusted for the cluster sampling design, unless the design effect was ≤1, when binomial exact confidence intervals are presented. The area under the receiver operating characteristics curve (AUC) was estimated from the logistic regression model. We compared our TB prevalence data with the results of TB prevalence surveys and a population level TB screening study that were published between 2000 and 2010 as identified trough searching PubMed and reference lists of survey reports, reviews [1] and reports. [4], [21] Publications were included if sensitivity and specificity of symptom and/or CXR screening were reported, or could be calculated (including 95% confidence intervals), in which case possible effects of cluster design were not taken into account. Graphics were produced with the R-ggplot2 package. [22].

## Results

The distribution of TB symptoms and CXR abnormalities among study participants, suspects, and bacteriologically confirmed cases are shown in Table 2. Of the 7,342 (36%) suspects that were identified in the survey, 1,833 (25%) had only a positive symptom screen as defined by strategy 1, 1,490 (20%) had both a positive symptom screen and a CXR abnormality, and 3,852 (52%) had only a CXR abnormality. The combined symptom and CXR screening strategy used in the survey (strategy 10; see Table 1) had 100% sensitivity. Sputum microscopy on all participants did not yield additional suspects, but in our survey led to the identification of three symptomatic smear-positive participants who had not gone to the CXR location (and due to the organization of field procedures, had initially not provided sputum for culture).

**Table 2:** Prevalence of TB symptoms and radiographic abnormalities in study participants, suspects, and cases of pulmonary TB in the prevalence survey.

	All Participants		Suspects§		Cases		HIV-infected	Smear+
Presence of	*n*	*(%)*	*n*	*(%)*	*n*	*(%)*	n(%)¶ **	n(%)¶
	20,566		7,342		123		52/101 (51)	51 (41)
Cough								
≥2 weeks	2,264	(11)	2,264	(31)	64	(52)	36/56 (64)	37 (58)
8–13 days	317	(2)	317	(4)	4	(3)	2/3 (67)	2 (50)
1–7 days	5,973	(29)	1,913	(26)	26	(21)	11/20 (55)	9 (35)
None reported	12,006	(58)	2,846	(39)	29	(24)	3/22 (14)	3 (10)
Missing	6	(0)	2	(0)	0	(0)		
Productive Cough								
Yes	6,615	(32)	3,788	(52)	84	(68)	42/70 (60)	45 (54)
No	13,937	(68)	3,558	(48)	39	(32)	10/31 (32)	6 (15)
Missing	14	(0)	6	(0)	0	(0)		
Haemoptysis								
Yes	663	(3)	663	(9)	9	(7)	8/9 (89)	8 (89)
No	19,897	(97)	6,677	(91)	114	(93)	44/92 (48)	43 (38)
Missing	6	(0)	2	(0)	0	(0)		
Fever								
>1 week	984	(5)	873	(12)	21	(17)	12/15 (80)	12 (57)
≤1 week (7 days)	5,336	(26)	2,181	(30)	42	(34)	20/34 (59)	17 (40)
None reported	14,132	(69)	4,224	(58)	60	(49)	20/52 (38)	22 (37)
DNK (whether had fever or not)	106	(1)	61	(1)	0	(0)		
Missing	8	(0)	3	(0)	0	(0)		
Night sweats								
>1 week	1,369	(7)	1,082	(15)	25	(20)	14/21 (67)	14 (56)
≤1 week (7 days)	5,315	(26)	2,167	(30)	42	(34)	28/37 (76)	17 (40)
None reported	13,766	(67)	4,031	(55)	56	(46)	10/43 (23)	20 (36)
DNK (whether had night sweats or not)	108	(1)	59	(1)	0	(0)		
Missing	8	(0)	3	(0)	0	(0)		
Weight loss; with change of fit of clothes								
Yes; Yes	4,016	(20)	2,099	(29)	68	(55)	34/53 (64)	34 (50)
Yes; No or DNK change of fit	1,578	(8)	643	(9)	9	(7)	3/7 (43)	3 (33)
No weight loss	14,296	(70)	4,324	(59)	42	(34)	12/37 (32)	13 (31)
DNK (whether had weight loss or not)	670	(3)	274	(4)	4	(3)	3/4 (75)	1 (25)
Missing	6	(0)	2	(0)	0	(0)		
Any symptom of any duration/severity*‡								
Yes	13,989	(68)	6,017	(82)	111	(90)	50/90 (56)	49 (44)
No	6,577	(32)	1,325	(18)	12	(10)	2/11 (18)	2 (17)
Study symptom screening algorithm† ‡								
Yes	3,490	(17)	3,481	(47)	75	(61)	40/62 (65)	40 (53)
No	17,076	(83)	3,861	(53)	48	(39)	12/39 (31)	11 (23)
CXR reading by clinical officer								
Any abnormality - of which	5,342	(26)	5,342	(73)	113	(92)	47/95 (49)	47 (42)
Pulmonary abnormality and/or pleural effusion	4,801	(23)	4,801	(65)	111	(90)	47/93 (51)	46 (41)
Other abnormality only	541	(3)	541	(7)	2	(2)	0/2 (0)	1 (50)
Normal	13,874	(67)	1,833	(25)	7	(6)	4/4 (100)	1 (14)
No CXR made	1,350	(7)	167	(2)	3	(2)	1/2 (50)	3 (100)
Study screening methods‡								
symptom screen positive; CXR abnormal	1,490	(7)	1,490	(20)	65	(53)	35/56 (63)	36 (55)
symptom screen positive; CXR normal	1,833	(9)	1,833	(25)	7	(6)	4/4 (100)	1 (14)
symptom screen positive; CXR missing	167	(1)	167	(2)	3	(2)	1/2 (50)	3 (100)
symptom screen negative; CXR abnormal	3,852	(19)	3,852	(52)	48	(39)	12/39 (31)	11 (23)
symptom screen negative; CXR normal	12,041	(59)	0		0			
symptom screen negative; CXR missing	1,183	(6)	0		0			

TB = pulmonary tuberculosis; CXR = chest radiograph DNK = does not know Smear+  =  sputum smear positive

† the presence of cough for more than 7 days, and/or two out of three of fever (present for >7 days), night sweats (present for >7 days).

§ A suspect is a survey participant with either a CXR abnormality (any abnormality) reported during field reading by clinical officers and/or symptoms suggestive of TB and/or a positive sputum smear microscopy result.

¶ Row percentage. All other percentages are column percentage. *including weight loss regardless of change in fit of cloths.

** The number and % with HIV-positive status out of the number with a known HIV status.

‡ In symptom-combinations a missing value or 'DNK' for symptom questions are considered negative.

All symptoms included in the screening questionnaire were significantly associated with PTB in single variable analysis, but in multiple logistic regression analysis only CXR abnormalities, cough and weight loss were independent predictors (Table 3). Cough for 2 or more weeks (strategy 1), which is a commonly used symptom screen in surveys, clinical practice and active case finding, had 52% sensitivity (95%CI 41–63) and 89% (95%CI 88–90) specificity (Table 4). The most sensitive symptom screen was the report of at least one symptom of any duration or severity (strategy 2); this symptom screen had 90% (95%CI 84–95) sensitivity and 32% (95%CI 30–34) specificity. Of 19,216 (93%) participants with a CXR, 5,342 (26%) had an abnormality, which had 94% (95%CI 88–98) sensitivity and 73% (95%CI 68–77) specificity. In HIV-infected participants, the sensitivity of symptom combinations was higher, and the sensitivity of CXR abnormalities was lower (Table 4) compared to HIV-uninfected, although the 95% CI’s overlap. The symptom screen applied in the survey (strategy 6) was positive in 3,490 (17%) participants, and had 61% sensitivity (95%CI 50–72) and 83% (95%CI 82–85) specificity. The accuracy details of this and additional strategies are shown in Table S1. The specificity of the combined symptom and CXR screen (strategy 10) was 65% (95%CI 61–68). When narrowing down on this combination, excluding the systemic symptoms (fever, night sweats, weight loss) from the symptom screen (strategy 12) or using a duration of cough of at least ≥2 weeks (strategy 13) non-significantly increased specificity to 66%. Excluding the systemic symptoms decreased sensitivity non-significantly from 100% (95%CI 97–100) to 97% (95%CI 92–99). The HIV status of the TB cases who would consequently be missed was not available. Reading CXRs for pulmonary and pleural abnormalities only, rather than any abnormality, would have missed one smear-negative culture-positive asymptomatic HIV-negative case with a CXR with a cardiac abnormality only.

**Table 3:** Associations between symptoms in the screening questionnaire and bacteriologically confirmed TB (n = 20,560).

		Unadjusted Odds Ratio (95%CI)		p-value	Adjusted OR (95%CI)*		p-value
Cough							
	≥2 weeks	9.5	(6.2–14.5)	<.0001	3.9	(2.5–6.1)	<.0001
	8–13 days	4.2	(1.6–11.0)	0.004	3.1	(1.2–8.3)	0.025
	<7 days or none	1			1		
Productive Cough^†^							
	Yes	4.6	(3.2–6.6)	<.0001			
	No	1					
Haemoptysis							
	Yes	2.4	(1.3–4.5)	0.007			
	No	1					
Fever >7 days^§^							
	Yes	4.1	(2.5–7.0)	<.0001			
	No	1					
Night sweats >7 days**							
	Yes	3.6	(2.4–5.5)	<.0001			
	No	1					
Weight loss resulting in changed fit of cloths							
	Yes	5.2	(3.8–7.1)	<.0001	2.9	(2.1–4.1)	<.0001
	No	1			1		
CXR reading by clinical officer							
	Pulmonary/pleural abnormality	46.9	(24.6–89.5)	<.0001	32.3	(16.3–64.2)	<.0001
	Other abnormality	7.4	(1.5–35.7)	0.013	6.4	(1.3–31.2)	0.021
	Normal	1			1		
	No CXR	4.4	(1.1–17.3)	0.034	4.7	(1.2–18.0)	0.024

* Only variables that significantly contributed to the multiple logistic regression model were included in the final model.

CI = Confidence Interval CXR = chest radiograph †§**Due to missing values: *n = 20,522; †n = 20,452; §n = 20,450.

**Table 4:** Diagnostic value of Cough ≥2 weeks, any symptom, and CXR.

Screening strategy (*strategy # as in * *Table 1*)	TB cases with positive screen	Participants without TB with positive screen	Sensitivity(%) (95%CI*)	Specificity(%) (95%CI)	PPV(%) (95%CI)	AUC†
Total N	123	20,443				
*1.* Cough ≥2 weeks§	64	2,200	52 (41–63)	89 (88–90)	2.8 (2.0–3.2)	0.71
in HIV-positive	36		69 (56–83)			
in HIV-negative	20		41 (25–57)			
in HIV-unknown	8		36 (17–59)			
*2.* Any symptom of any duration or severity§	111	13,878	90 (84–95)	32 (30–34)	0.8 (0.6–1.0)	0.61
in HIV-positive	50		96 (87–100)			
in HIV-negative	40		82 (68–91)			
in HIV-unknown	21		95 (77–100)			
*8.* CXR – any abnormality**	113	5,229	94 (88–98)	73 (68–77)	2.1 (1.5–2.7)	0.83
in HIV-positive	47		92 (81–98)			
in HIV-negative	48		100 (93–100)			
in HIV-unknown	18		86 (68–100)			
*11.* Cough ≥2 weeks or any CXR abnormality§	119	8,702	97 (92–99)	57 (55–60)	1.4 (1.1–1.7)	0.79
in HIV-positive	52		100 (93–100)			
in HIV-negative	49		100 (93–100)			
in HIV-unknown	18		82 (60–95)			

CI = Confidence Interval CXR = Chest radiograph.

* Where the design effect was ≤1 CI’s were not adjusted for cluster design but binomial exact CI presented.

† AUC = Area under the receiver operating characteristic curve.

§ Denominator for HIV-positive n = 52, HIV-negative n = 49, HIV-unknown n = 48.

** 3 cases did not have a CXR, 1HIV+, 1HIV-, 1HIVunknown, so denominators are 120, 51, 48 and 21 respectively. For specificity: 1347 missing records.

The diagnostic accuracy, as summarized by the AUC, were similar for screening by CXR alone (0.83 for presence of any abnormality; 0.84 when considering pulmonary and pleural abnormalities only), and for combinations of CXR and symptoms (0.82–0.84). The negative predictive values (or post-test probability of not having TB with a negative screen), of all algorithms were between 99.6% (for cough ≥3weeks and haemoptysis) and 100%, and are not shown in the tables. The pre-test probability of not having PTB without application of any screening tool was 99.4%.

Applying sequential rather than parallel screening scenarios could potentially reduce the number needed to X-ray by 17%, if participants with a positive symptom screen would not undergo CXR. Performing CXR first would reduce the number needed to be interviewed by 33%. Sequential screening would result in only 2% reduction in cultures, but overestimate prevalence by 4%. CXR screening only would be more efficient, and reduce the number of suspects requiring sputum culture by 23%, would by definition not require interviews, and result in a prevalence change of <2% due to a smaller number of participants in the denominator, even though cases would be missed among participants who did not attend the CXR screening. Screening for ‘any symptom’ followed by CXR and a different algorithm depending on HIV status of the participant (strategy 16), would reduce the number requiring CXR evaluation and the number of suspects by approximately one third but underestimate prevalence by 11%.

**Figure 1:**
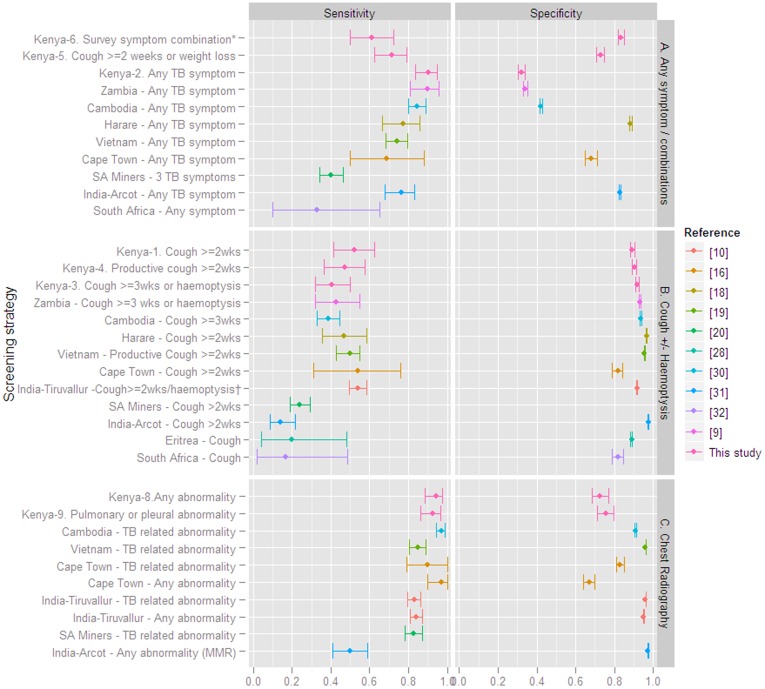
Sensitivity and specificity of symptom and chest radiography screening in population based TB screening (10 prevalence surveys [9], [10], [13], [16], [18], [19], [28], [30], [31], [32] including our report and 1 pre-mass IPT screening [**20**]), reported since the year 2000. Panel A summarizes screening for ‘any TB symptom’ or combinations of 3 or more symptoms. Panel B summarizes screening for cough of ≥2 or ≥3 weeks, and panel C summarizes chest radiography screening. *Kenya-1, Kenya-2 etc. refers to the strategy numbers described in Table 1 and Table 4. †includes also fever >1 month or chest pain. MMR = Mass Miniature Radiography.

The sensitivity and specificity of symptom and CXR screening algorithms reported from the recent literature are summarized in Figure 1 together with the sensitivity and specificity of symptom screening strategies 1–6 and CXR screening in our survey, showing a wider variation in the accuracy of symptom screening compared to CXR.

## Discussion

In our TB prevalence survey, CXR and combinations of CXR and symptoms were more sensitive and more accurate screening strategies for identifying TB cases than symptom screening alone. Performing sputum microscopy on all participants did not yield additional suspects.

Symptom screening alone is attractive because it is simple, does not require expensive equipment, and has been applied in several surveys. [6], [23], [24], [25] Some authors have proposed applying a correction factor [26] to adjust the prevalence estimate for the lower sensitivity. However, the large variation in the sensitivities of symptom screening strategies obtained in different surveys (Figure 1) limits the generalizability of a correction factor and the comparability of prevalence estimates obtained from different surveys. The reasons for the variation include differences in the number, duration, and severity of symptom combinations included in the questionnaire and in the screening criteria, repeatability of responses to symptom questionnaires [27], possibly cultural differences, and wide confidence intervals. The comparison between studies is further limited by different reference standards (positive culture or microscopy [9], [10], [16], [20], microscopy only [28], or confirmation by a subsequent culture and/or clinical disease at follow up [20], [29]). Estimates from surveys where sputum of all participants was cultured [9], [16], [29], would be more reliable, but these are few, and ascertainment bias in sensitivity estimates obtained from surveys with a suspect screening step [10], [19] will increase if a less sensitive screening tool is applied. Nevertheless the sensitivity of cough ≥3 weeks (with or without haemoptysis) was very similar in the studies from Cambodia [30], Zambia [9], and Kenya (strategy #3). Similarly, the reported sensitivity of a 2-week cough (or productive cough) screen was approximately 45–55% in the studies from India [10], Cape Town [16], Harare [18], Vietnam [19], and Kenya (strategies 1 and 4), although a wide range of sensitivities has been reported. [20], [31], [32] Due to the lower sensitivity, symptom screening alone is less preferred for prevalence surveys. By contrast, for active case finding, a less sensitive but frequently used case finding tool has been shown to reduce prevalence [29], and may be more effective in reducing transmission than a less frequently used highly sensitive tool [33], and symptom screening may thus be applicable for active case finding. A systematic review and possibly a meta-analysis may be useful in standardizing symptom questionnaires, if differences in symptom definitions, ascertainment bias due to suspect selection methods, and reference standards are accounted for in the analysis.

In this study screening for ‘any TB symptom’ had high sensitivity (90%), especially in HIV-infected (96%), but very low specificity, consistent with other prevalence surveys from African populations with high HIV prevalence [9], [18], [34], Cambodia [30] (Figure 1), and also with the algorithm that was developed for TB screening among HIV-infected individuals in a clinical setting [35]. The scenario to first screen for any symptom, followed by CXR and HIV status may be more applicable to active TB case finding strategies in high HIV prevalence populations, if followed by more specific tests. The lower sensitivity of the algorithm in the HIV-negative population, 82% (95% CI 68–91) would result in more missed HIV-negative TB cases, which for case finding is less of a concern. Due to on average slower disease progression, those missed cases may be found at a next round, or self cure, while among HIV-infected the mortality risk from undiagnosed TB is high. Active case finding targeting smear-positive TB cases with a cough of 2 weeks or more has been shown to reduce transmission [29], although the sensitivity in our study is low.

For prevalence surveys, the still considerable variation in the sensitivity of the criteria ‘any symptom’ in different studies, and the lower sensitivity in HIV-uninfected compared to HIV-infected are concerns for accurate prevalence estimates.

CXR screening had higher sensitivity and overall accuracy in our study compared to symptom screening alone. In the other reports included in Figure 1, sensitivity was also high and varied less, with the lower values originating from a study using fluorographs (mass miniature radiography). [10] CXR screening alone has been applied in some surveys [8] and been proposed by others. [16] Screening for presence of any abnormality on CXR alone in our study would have reduced the number of cultures by a quarter with only a small underestimation of the prevalence. More standardization of the type of abnormalities that are considered a positive screen may be useful. We choose to use any abnormality to avoid interpretation of which abnormalities would qualify by the field readers, who were not expert readers. Most studies only included abnormalities consistent with TB, which require expert reading, and have lower sensitivity and repeatability than presence of any abnormality. [2], [15], [36] The lower sensitivity of CXR in HIV-infected found in this and other studies [37], [38] is however a concern since HIV prevalence is high in several of the focus countries for TB prevalence surveys. [2].

A simple and inexpensive test to identify a biomarker would eliminate the need for symptom or CXR screening, but is currently not available. The utility of the Xpert® MTB/RIF assay [39] (Cepheid, Sunnyvale, CA USA) for prevalence surveys requires further evaluation. In the interim, a combined symptom and CXR screening remains the preferred methodology [1], [21], in order not to compromise prevalence estimates.

The specificity of CXR and symptoms screening combined was 65% in our survey but reached over 90% in other studies. [19], [20], [40] Small gains in specificity could be achieved by changing the cough or CXR criteria in our screening algorithm. A simpler symptom combination, like cough ≥2 weeks and/or weight loss (strategy #5), the strongest predictors in logistic regression, had high sensitivity, but lower specificity compared to the survey strategy (strategy #10), and would reduce the efficiency of screening.

The reduction of the numbers of cultures, CXRs and/or interviews required that may be achievable by sequential screening are modest, and should be balanced against the disadvantages of not having standardized procedures for all participants, which may lead to more errors. Also, if information on other risk factors, like socio-demographic information, is collected through questionnaires [41], omitting questions on TB symptoms for some participants will not necessarily reduce resource needs. With increasing availability of digital X-ray and field reading by non-experts trained on chest radiograph interpretation and supervised during the study, or in the future a reading-software [42], the implications of a 10% change in the number of CXRs would be rather minimal.

Limitations of our study include that we performed only one culture, while two or more would have yielded more cases. [43] We did not perform cultures on all participants, which would have allowed for the identification of subclinical TB [9], [32], [44], i.e. positive sputum cultures in persons without symptoms and or CXR abnormalities [17], [37]. Because of this ascertainment bias, our sensitivity estimates and negative predictive values are overestimated, however we estimate this to be by less than 5%. [16], [17] In a survey from Zambia with similar HIV prevalence, one sputum culture was done on all participants, but not CXRs. However the sensitivity of ‘any symptom’ and of cough>3wks in the Zambian and our survey are very similar (Figure 1). We obtained HIV status only on identified cases. HIV status on all participants, or a random sample, would have allowed for calculation of specificity and predictive values of symptoms and CXR screening by HIV status. Although both CXR and culture require expensive equipment, technical expertise, and quality assurance interventions, in many settings where national TB prevalence surveys are recommended, acquiring the capacity for CXR screening will be easier and faster achieved than scaling up TB culture laboratory capacity to accommodate the number of cultures needed for large population surveys. CXR equipment can be transported to the participants, becomes potentially simpler with digital equipment, and radiographic technologists are readily available in African countries.

In conclusion, a combination of CXR screening and symptom screening remains an important methodology to identify suspects in TB prevalence surveys where the examination of sputum of all participants by mycobacterial culture or tests of equivalent sensitivity is not feasible. Symptom screening alone has value for TB case finding.

## Supporting Information

Table S1 **Additional screening strategies to those reported in** **Table 4** **, either for comparison with strategies in other surveys/guidelines, or simplifications from the survey combination.** CI = Confidence Interval; CXR = Chest radiograph; §AUC = Area under the receiver operating characteristic curve; *Where the design effect was ≤1 CI’s were not adjusted for cluster design but binomial exact CI presented. **3 cases did not have a CXR, so gold standard = 120 for sensitivity. For specificity: 1347 missing records. † the presence of cough for more than 7 days, and/or haemoptysis or two out of three of fever (present for >7 days), night sweats (present for >7 days), weight loss resulting in a changed fit of clothes. See also Table 1.
(DOC)
