# Supplementary material for: Screening Strategies for Tuberculosis Prevalence Surveys: The Value of Chest Radiography and Symptoms
Source: PLoS One. 2012 Jul 6;7(7):e38691. doi: 10.1371/journal.pone.0038691 (PMC3391193; doi:10.1371/journal.pone.0038691)
Supplement: Table S1 — Additional screening strategies to those reported in Table 4 , either for comparison with strategies in other surveys/guidelines, or simplifications from the survey combination. CI = Confidence Interval; CXR = Chest radiograph; §AUC = Area under the receiver operating characteristic curve; *Where the design effect was ≤1 CI’s were not adjusted for cluster design but binomial exact CI presented. **3 cases did not have a CXR, so gold standard = 120 for sensitivity. For specificity: 1347 missing records. † the presence of cough for more than 7 days, and/or haemoptysis or two out of three of fever (present for >7 days), night sweats (present for >7 days), weight loss resulting in a changed fit of clothes. See also Table 1. (DOC) [file pone.0038691.s001.doc]

Table S1. Additional Screening strategies to those reported in Table 2, Either for comparison with strategies in other surveys/ guidelines, or simplifications from the survey combination.

|  | TB cases with positive screen | Participant without TB with positive screen | Sensitivity(%) (95%CI*) | Specificity(%) (95%CI) | PPV(%)  (95%CI) | AUC^§^ |
| --- | --- | --- | --- | --- | --- | --- |
|
| Total N | 123 | 20,443 |  |  |  |  |
| Resembling strategies used in other studies | | | | | | |
| *3*. Cough ≥ 3 weeks or haemoptysis | 50 | 1,694 | 41 (32-50) | 92 (91-93) | 2.9 (2.1-3.8) | 0.66 |
| *4*. Productive cough ≥ 2 weeks | 58 | 1,987 | 47 (37-58) | 90 (89-91) | 2.8 (2.0-3.6) | 0.69 |
| *7.* Cough ≥ 2 weeks and smear positive (≥ scanty [1-9 AFB/whole smear]) | 37 | 2 | 30 (20-40) | 100 (100-100) | 95 (83-99) | 0.65 |
| Simplifications from survey combination | | | | | | |
| *6*. Symptom screening combination used in this survey^†^ | 75 | 3,415 | 61 (50-72) | 83 (82-85) | 2.1 (1.6-2.7) | 0.72 |
| *5*. Cough ≥ 2 weeks or weight loss | 88 | 5,543 | 72 (63-79) | 73 (71-75) | 1.6 (1.2-2.0) | 0.72 |
| *10.* Screening strategy used in this survey: any abnormality on CXR and/or positive symptom screening combination 1. | 123 | 7,219 | 100 (97-100) | 65 (61-68) | 1.7 (1.3-2.1) | 0.82 |
| *9.* CXR – pulmonary and/or pleural abnormality only** | 111 | 4,690 | 93 (86-97) | 75 (71-80) | 2.3 (1.7-3.0) | 0.84 |
| *12*. Any abnormality on CXR and/or cough >7 days (systemic symptoms excluded) | 119 | 6,872 | 97 (92-99) | 66 (63-70) | 1.7 (1.3-2.1) | 0.82 |
| *13*. Any abnormality on CXR and/or cough ≥ 2 weeks and/or ≥2 out of fever (for > 7 days), night sweats (for >7 days), weight loss(changed fit of clothes) | 123 | 7,042 | 100 (97-100) | 66 (62-69) | 1.7 (1.3-2.1) | 0.83 |
| *14*. Pulmonary and/or pleural abnormality on CXR and/or cough ≥ 2 weeks and/or ≥2 out of fever (for > 7 days), night sweats (for > 7 days), weight loss (changed fit of clothes) | 122 | 6,594 | 99 (96-100) | 68 (64-71) | 1.8 (1.4-2.2) | 0.84 |

CI=Confidence Interval CXR=Chest radiograph

§AUC=Area under the receiver operating characteristic curve

*Where the design effect was ≤1 CI’s were not adjusted for cluster design but binomial exact CI presented.

** 3 cases did not have a CXR, so gold standard=120 for sensitivity. For specificity: 1347 missing records

† the presence of cough for more than 7 days, and/or haemoptysis or two out of three of fever (present for > 7 days), night sweats (present for > 7 days), weight loss resulting in a changed fit of clothes. See also text Box
